# Characterization and low-cost preservation of *Chromobacterium violaceum* strain TRFM-24 isolated from Tripura state, India

**DOI:** 10.1186/s43141-021-00241-z

**Published:** 2021-10-01

**Authors:** Sushil K. Sharma, Rakhi Dhyani, Ees Ahmad, Pankaj K. Maurya, Madhu Yadav, Ramesh Chandra Yadav, Vinod Kumar Yadav, Pawan K. Sharma, Mahaveer P. Sharma, Aketi Ramesh, Anil K. Saxena

**Affiliations:** 1 National Agriculturally Important Microbial Culture Collection (NAIMCC), ICAR-National Bureau of Agriculturally Important Microorganisms, Kushmaur, Maunath Bhanjan, Uttar Pradesh 275 103 India; 2 Present Address: ICAR-National Institute of Biotic Stress Management, Baronda, Raipur, Chhattisgarh 493 225 India; 3 ICAR-Indian Institute of Soybean Research, Khandwa Road, Indore, Madhya Pradesh 425 001 India

**Keywords:** *Chromobacterium*, Natural plant gums, Preservation

## Abstract

**Background:**

*Chromobacterium* species, through their bioactive molecules, help in combating biotic and abiotic stresses in plants and humans. The present study was aimed to identify, characterize and preserve in natural gums the violet-pigmented bacterial isolate TRFM-24 recovered from the rhizosphere soil of rice collected from Tripura state.

**Results:**

Based on morphological, biochemical and 16S rRNA gene sequencing, the isolate TFRM-24 was identified as *Chromobacterium violaceum* (NAIMCC-B-02276; MCC 4212). The bacterium is saprophytic, free living and Gram negative. The strain was found positive for production of IAA, cellulase, xylanase and protease, and showed tolerance to salt (2.5%) and drought (-1.2 MPa). However, it showed poor biocontrol activity against soil-borne phytopathogens and nutrient-solubilizing abilitiets. *C. violaceum* strain TRFM-24 did not survive on tryptic soya agar (TSA) beyond 12 days between 4 and 32 °C temperature hence a method of preservation of this bacterium was attempted using different natural gums namely *Acacia nilotica* (*babul*), *Anogeissus latifolia* (*dhavda*), *Boswellia serrata* (*salai*) and *Butea monosperma* (*palash*) under different temperature regime (6–32 °C). The bacterium survived in *babul* gum (gum acacia), *dhavda* and *salai* solution at room temperature beyond a year.

**Conclusion:**

Based on polyphasic approach, a violet-pigmented isolate TRFM-24 was identified as *Chromobacterim violaceum* which possessed some attributes of plant and human importance. Further, a simple and low-cost preservation method of strain TRFM-24 at room temperature was developed using natural gums such as *babul*, *dhavda* and *salai* gums which may be the first report to our knowledge.

**Supplementary Information:**

The online version contains supplementary material available at 10.1186/s43141-021-00241-z.

## Background

*Chromobacterium violaceum* is abundantly found in soil and water ecosystems of tropical and subtropical regions of the world. It was first reported by Boisbaudran in 1882 [1]. The bacterium produces a characteristic purple pigment called ‘violacein’ (C_20_H_13_N_3_O_3_) which consists of 5-hydroxyindole, a α-pyrrolidone and an oxindole unit, formed from the condensation of two modified tryptophan molecules [2]. Many *Chromobacterium* species have been reported from different niches (Supplementary Table 1). Besides, a new bacterial species *C. suttsuga* NRRL B-30655 having insecticidal property is distinct from all other *Chromobacterium* species described earlier [3]. *C. violaceum* is a saprophytic, pathogenic or non-pathogenic, free-living, facultative anaerobic, motile, oxidase-positive bacillus and Gram-negative bacteria belonging to the *Neisseriaceae* family of Betaproteobacteria. *C. violaceum* has potential use in agricultural, medical, industrial and biotechnology fields, including control of plant diseases caused by phytopathogens and insect pest [4, 5] infections and diseases in humans [6–8]; prevention of transmission of diseases by mosquitoes *Anopheles gambiae* and *Aedes aegypti* [9]; hydrogen cyanide-mediated gold recovery from electronic waste [10]; degradation of hydrocarbon and phenol [11, 12] and production of antitumoural, antiviral, anti-*Plasmodium*, antibacterial and anti-leishimanial substances [13–18]; and solubilization of gold [19] production of biopesticidal molecules and chitinolytic enzymes [20, 21]. Recently, Ahmad et al. [22] used *C. violaceum* TRFM-24 as an indicator for detecting tryptophan in the tris-minimal medium supplemented with acid hydrolysed casein hydrolysate to confirm production of indole-3-acetic acid (IAA) by the tryptophan-independent pathway operating in *Micrococcus aloeverae* DCB-20.

Around 4000 strains of *C. violaceum* have been reported from various niches across the globe. In India, 30 strains of *C. violaceum* have been reported from soil, water, roots, leaves, and tissues of plants and animals collected from Goa, Tripura, Kerala, Tamil Nadu, Orissa, Gujarat, Manipur and Maharashtra states. We isolated a putative *Chromobacterium* spp. from Tripura state which was found to be sensitive to low temperature and does not survive for more than 10 days on the culture medium. Although long-term storage by lyophilization has been well known, its application becomes cumbersome due to its high cost and requiring technology-oriented and professional laboratory staff. Thus, most of the laboratories maintain *C. violaceum* cultures by regular sub-culturing within a week period [23]. Therefore, it is important to develop a cost-effective method for stable and long-term preservation of this bacterium to ensure maintenance of its viability and genetic stability considering its multifaceted uses as mentioned above. Cryoprotectants such as glycerol, trehlose, polyvinypyrrolidone, sucrose, skim milk, DMSO and methanol are available for long-term preservation of many bacterial cultures, but these are generally expensive. Recently, use of low-cost natural substances like natural polymers particularly gum acacia and pullalan that are nontoxic and soluble in water have been used for preservation of *Bacillus subtilis, B. anthracis, Staphylococcus aureus* and *E. coli* [24, 25]. Natural gums (gums from plants) are hydrophilic carbohydrate polymers of high molecular weights, composed of monosaccharide units joined by glucosidic bonds. These gums are either soluble in water or absorb water and swell up or disperse in cold water to give a viscous solution or jelly. On hydrolysis, they yield carbohydrates such as arabinose, galactose, mannose and glucuronic acid [26]. However, use of these gums in preservation of *Chromobacterium* sp. has not been investigated. Hence, the aim of the present study was to (1) identify pigmented bacterium isolated from the rhizosphere soil of Tripura state, (2) characterize it functionally to reveal its plant growth-promoting traits and (3) develop a low-cost and simple method to preserve the *C. violaceum* TRFM-24 for a considerable period of time without losing its viability and stability.

## Methods

### Sampling for isolation of bacterial isolate TRFM-24

Twenty-one soil samples including 16 rhizosphere soil of crops and forest trees were collected at 0–20 cm depth from twenty-one  locations spread across Tripura state of India during February, 2019 (Supplementary Fig.1). Of these locations, soil sampling was carried out from Swarna Masoori rice–harvested field located at Fatikcherra village (Mohanpura) of West Tripura district (N 23° 58.321 E 91° 22.489 with altitude 19 MSL). Mean day and night temperature during February, 2019 ranged from 16 to 28°C. All the collected soil samples were kept in zipper-lock polyethene bags and kept at 4 °C in the refrigerator for 3 days until transported to ICAR-National Bureau of Agriculturally Important Microorganisms (ICAR-NBAIM), Maunath Bhanjan, Uttar Pradesh, India. The soil sample of Fatikcherra was diluted serially in 0.85% saline solution and plated on Angle’s agar nonselective medium [27] followed by incubation at 28 °C for 48–72 h for appearance of colonies of bacteria. At the same time, the remaining soil samples were also processed for isolation of bacteria. The soil characteristics of Fatikcherra are pH 5.2, organic carbon 1.85%, N 108 μg g^-1^ soil, P 8.9 μg g^-1^ soil, Zn 0.65 μg g^-1^ soil, Fe 25.63 μg g^-1^ soil, Mn 33.88 μg g^-1^ soil and Cu 1.45 μg g^-1^ soil.

### Phenotypic, biochemical and fatty acid methyl ester characterization

Of all the bacterial isolates recovered, only one unique violet coloured isolate was isolated and selected for further study. The isolate was designated as a TRFM-24 and cultivated on different media such as tryptic soya agar (TSA), nutrient agar (NA), Kings B agar (KB agar), brain heart infusion (BHI) agar, MacConkey agar and Luria Bertani (LB) agar media and incubated at 28 ± 2 °C to study the colony morphology and variation in pigmentation. Cell morphology, motility and Gram’s reaction of the isolate were assessed by using standard methods [28, 29]. Blood agar medium supplemented with 5% human blood was used for haemolysis test: a clear or semi-clear zone around the colony indicated a positive test. DNAase production by isolate was carried out using DNAase test agar base (HiMedia, Mumbai, India). Appearance of clear zone on flooding with 1M HCl around bacterial colony is indicative of DNAase production [30]. Growth at pH values (4.0, 5.0, 5.5, 6.0, 6.5, 7.0, 8.0, 9.0, 10.0, 11.0, 12.0) was assessed using TSA and TSB as basal medium based on the requirements. Acid production from carbohydrates and other biochemical parameters such as catalase and oxidase test, nitrate reduction, hydrogen sulfide (H_2_S) production, pigmentation under anaerobic condition, gelatine liquefaction, urea hydrolysis, Simmon citrate utilization, triple sugar agar utilization and indole production were studied using standard methods [31]. Amino acid utilization by the bacterium was performed using amino acids such as arginine, ornithine and lysine. The bacterial isolate TRFM-24 was characterized based on the extraction of whole-cell fatty acids of the bacterial isolates derivatised to methyl esters and analysed by gas chromatography (GC) using the Sherlock Microbial Identification System (MIDI, Inc., Newark, DE, USA) [32, 33].

### Identification of TRFM-24 by 16S rRNA gene sequencing 

DNA extraction and amplification of 16S rRNA gene of the isolate TRFM-24 was carried out using the method of Henry et al. [34] and was sequenced from Eurofins, Kochi, India. Phylogenetic neighbours and the calculation of pairwise 16S rRNA gene sequence similarities were achieved using the EzTaxon server. The 16S rRNA gene sequence of the isolate TRFM-24 and the members of closely related genera was retrieved from the EzTaxon server [35] and aligned using CLUSTAL W in MEGA version 7 [36]. The neighbour-joining–based phylogenetic tree was reconstructed using standard parameters of the CLUSTAL W alignment. Evolutionary analysis was carried out using MEGA 7. The topology of the evolutionary tree was evaluated by a bootstrap analysis [37] of the neighbour-joining method based on 1000 replicates using the MEGA 7 software. The processed nucleotide sequence data with its identity was submitted in the NCBI GenBank sequence database to acquire accession number. Finally, the identified bacterium *Chromobacterium violaceum* strain TRFM-24 (GenBank: MK841034) was deposited in two collections namely National Agriculturally Important Microbial Culture Collection (NAIMCC; World Data Centre for Microorganisms (WDCM) No 1060; http://www.wfcc.info/ccinfo/collection/by_id/1060; an International Depository Authority (IDA)), ICAR-NBAIM, Mau, Uttar Pradesh, India and National Centre for Microbial Resource (MCC; WDCM 930 http://www.wfcc.info/ccinfo/collection/by_id/930; an IDA), Pune, Maharashtra, India with accession numbers NAIMCC-B-02276 and MCC 4212, respectively.

### Functional characterization

The bacterial strain TRFM-24 was further characterized for different functional traits such as production of indole-3-acetic acid (IAA), siderophore and ACC deaminase, solubilization of zinc, phosphorus and potassium; and antagonism against phytopathogens using standard procedures. Bacterial strain was tested for IAA production by the method as described by Brick et al. [38]. Siderophore production assay was performed on the Chrome Azurol S (CAS) agar medium incubated for 72 h at 28 ± 2 °C. The development of yellow-orange halo around the bacterium was indicative of siderophore production [39]. Zinc and phosphate solubilization were assayed on Tris minimal-yeast extract agar medium supplemented separately with 0.1% Zn as zinc oxide, zinc phosphate and zinc carbonate (insoluble zinc source), 0.5% tricalcium phosphate as insoluble phosphorus source, and 0.5% potassium aluminium silicate as potassium source [40–42]. The 1-aminocyclopropane-1-carboxylic acid (ACC) deaminase activity was performed on Dworkin Foster (DF) medium supplemented with ACC as described by Govindaswamy et al. [43]. HCN production was determined by the qualitative method of Kremer and Souissi (2001) [44]. Antagonistic test was performed using dual plate technique against *Rhizoctonia solani*, *Macrophomina phaseolina* (clusterbean), *Sclerotium rofsii, Colletotrichum gloeosporioides* (NAIMCC-F-02704), *F. oxysporum* f. sp. *lycopersici* (NAIMCC-F-00892), *F. clamydosporium (*NAIMCC-F-00769), *F. irregular*, *F. equiseti, F. udum (*NAIMCC-F-01047*), F. verticilloides* (NAIMCC-F-03973) *and Curvularia geniculata* on PDA + NA medium. Tolerance to abiotic stresses such as salinity and drought was also assessed. Salinity tolerance was assessed by growing the bacterium on TSA supplemented with different concentrations of sodium chloride (2, 4, 6, 7, 8, 9, and 10%) followed by incubation at 28 ± 2 °C for 96 h. The growth of bacterium at particular concentration was indicative of its tolerance level. Tolerance to moisture stress was also analysed by growing the bacterium in nutrient broth supplemented separately with PEG-6000 at concentrations of 5, 8, 9.3, 15, 20, 30% equivalent to osmotic potentials (-0.453, -0.950, -1.20, -2.77, -4.64, and -9.802 MPa (megapascal) respectively followed by incubation at 28 ± 2 °C for 96 h [45].

### Viability test of TRFM-24

Viability and growth of isolate TRFM-24 was evaluated *in vitro* on TSA. Twenty-four-hour–grown active TRFM-24 culture was streaked on 9 plates of TSA and further grown for 24 h in a biological oxygen demand (BOD) incubator at 28 ± 2 °C. Later on, 3 plates each were incubated in the BOD incubator (28 ± 2 °C), cold room (6–8 °C) and room temperature (26–32 °C) for its growth, pigmentation and survival. The culture from all the 9 plates incubated previously under 3 different conditions was subsequently re-streaked on fresh medium to observe survival and growth. The growth of the bacterium was observed on every second day until no growth was observed on the plates (up to 12 days).

### Preservation of TRFM-24

The strain TRFM-24 was evaluated *in vitro* for its viability and long-term preservation using natural gums. The *in vitro* experiment consisted of four gums namely *Acacia nilotica* (L.) Wild. ex Delile [*babul*]*, Anogeissus latifolia* (DC.) Wallich ex Guill. *&* Perr. [*dhavda*], *Butea monosperma* (Lam.) kuntze [*palash*] and *Boswellia serrata* Roxb [*salai*] that were procured from different parts of Madhya Pradesh state of India. The survival of *Chromobacterium* was examined in four gums at different time intervals under three conditions viz. BOD incubators (28 ± 2 °C), cold room (6–8 °C) and room temperature (26–32 °C) in a 4 × 3 factorial CRD design with three replications. A 1.5% aqueous light viscous solution of each gum was prepared by dissolving 1.5 g gum in 100 ml hot tap water (pH 6.8; 50 °C) in borosilicate amber bottle followed by filtration in tea filter to remove debris, if any. An aliquot of 1.5 ml viscous solution of each gum was poured in a 2-ml capacity Eppendorf tube. One hundred and eight (108) tubes for each gum were prepared. A total of 432 tubes of four gums (108 tubes × 4 gums) were autoclaved at 121 °C twice after a 24-h interval in order to kill spore-forming microbes. After sterilization, all the tubes were kept at 4 °C until further utilization. The bacterial suspension was prepared by growing the strain TRFM-24 in 100 ml of TS broth for 48 h at 28 ± 2 °C, followed by centrifugation at 5000 rpm for 10 min to form pellet of bacterial cells. The pellet was washed twice with sterile distilled water followed by preparation of bacterial suspension of 1 optical density (OD) (620 nm) in sterile distilled water. The population count of suspension was 10^12^ CFU ml^-1^. Each tube containing 1.5 ml gum solution was inoculated with 200 μl of bacterial suspension of 1 OD. Out of 108 tubes for each gum, 3 lots of 36 tubes each were incubated in three different conditions: (1) BOD incubator, (2) cold room and (3) room temperature up to 360 days. In addition to the above methods, the strain TRFM-24 was also kept in 16% glycerol stock and stored in deep freezers at – 20 °C and − 80 °C and lyophilized in skimmed milk in order to observe the viability. The viability test and population of bacterial culture was observed at an interval of 30 days for around 360 days. To test the viability, 10 μl aliquot from each vial was spotted on TSA followed by incubation at 28 ± 2 °C for 48 h to observe growth of the bacterium. Growth of the bacterium on TSA was indicated positive for viability, whereas no growth indicated negative in the test. Similarly, population of bacterium from each vial was also enumerated on TSA using appropriate dilution.

### Statistical analysis

The population (CFU ml^-1^) was transformed into log value (log CFU ml^-1^) which was subjected to statistical analysis. The analysis was carried out using SAS statistical software (ver.9.2; SAS Institute., Cary, NC, USA). One-way analysis was done using the analysis of variance (ANOVA) procedure in SAS enterprise guide 4.2, and the Fisher least significant differences (LSD) and Tukey’s test were used to separate the treatment means. Two-way analysis was also carried out to differentiate between the method of storage and time intervals among each gum and three-way analysis to determine the differences between method of storage, gums and time intervals.

## Results

More than 500 diverse, pigmented and non-pigmented bacteria were isolated from 21 samples. A dark violet–coloured bacterial colony from the soil sample of Fatikcherra appeared on the Angle’s agar plate. This pigmented colony was picked up and re-streaked until a visible uniform culture appeared. The isolate was designated as TRFM-24. Out of the 21 samples, isolate TRFM-24 was recovered only from one soil sample. The culture of TRFM-24 was maintained on this medium after regular sub-culturing at an interval of 7–10 days. The culture was lyophilized using skimmed milk for its long-term preservation in order to maintain originality of culture because sub-culturing at frequent intervals induces variability in traits with loss of typical pigmentation.

### Identification and functional  characterization

**Fig. 1:**
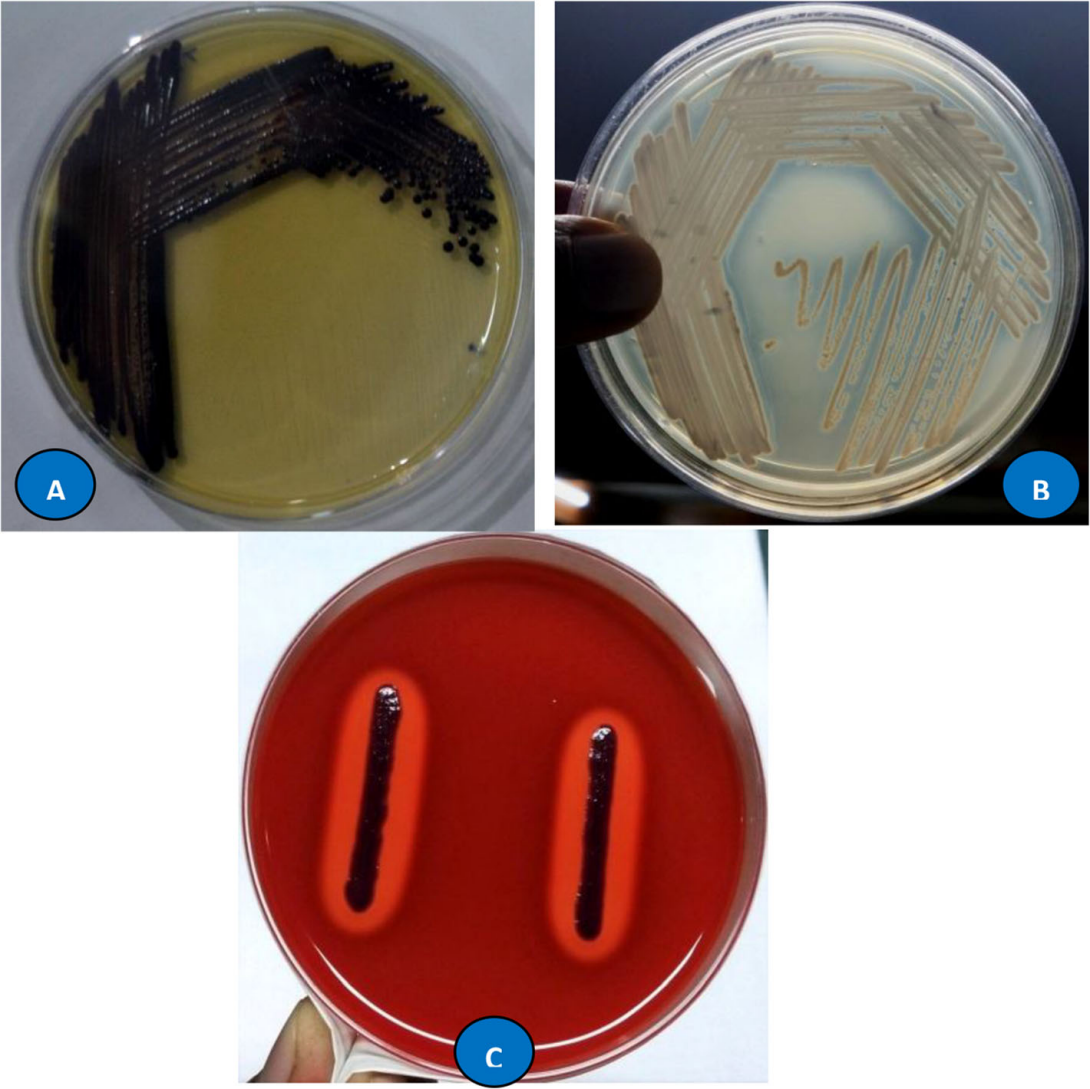
Colony morphology (**A**), DNAase test (**B**) and haemolytic test (**C**) of *Chromobacterium violaceum* strain TRFM-24

**Fig. 2:**
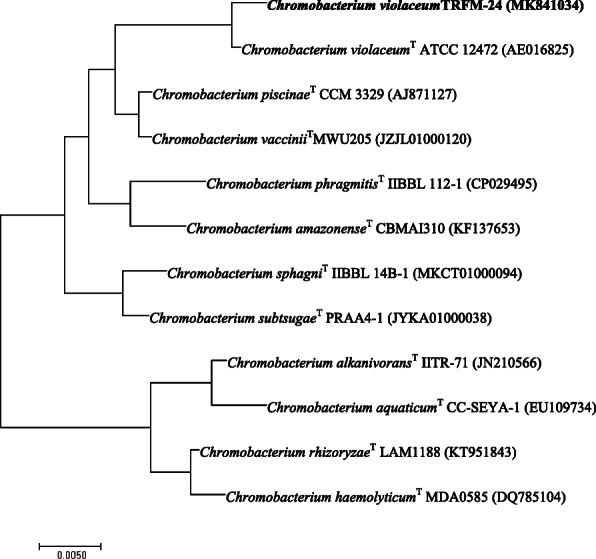
Neighbour-joining phylogenetic tree constructed on the basis of 16S rRNA gene sequences of *C. violaceum* strain TRFM-24 and other species of *Chromobacterium*. The evolutionary tree was constructed using MEGA 7

The isolate was characterized morphologically and biochemically including FAME’s profiling and 16S rRNA gene sequencing methods, and results are given in Table 1 & Fig.1). Phylogenetic analyses based on 16S rRNA gene sequence indicated close relation of isolate TRFM-24 to *Chromobacterium violaceum* ATCC (American Type Culture Collection) 12472 (Fig. 2). As far as functional characteristics are concerned*,* the strain TRFM-24 was found to be positive for production of IAA, cellulase, xylanase, protease and ammonium, and negative for siderophore production, HCN and ACC deaminase. It did not solubilise P, Zn and K under *in vitro* conditions. The strain grew well at temperature 28–30 °C, but no growth occurred at 10 °C and 45 °C and could tolerate sodium chloride salt up to 2.5% and withstood -1.2 MPa (9.3% PEG 6000). The strain did not show any antagonism toward test phytopathogens used in this study (Table 2).

**Table 1:** Phenotypic and molecular characteristics of *Chromobacterium vialoceum* strain TRFM-24

Characteristics	Strain TRFM-24
Colony feature after 24-h growth	Growth on TSA, BHI, MacConkey agar, NA: Light purple, circular, smooth, entire, convex, size 0.5 mm and non-flourescent KB agar: pin point colony with no pigmentation; LB agar: dark purple, circular, smooth, entire, convex, size 1.0 mm and non-flourescent
Cell shape	rod
Gram reaction	**-**ve
Catalase test	**+**ve
Oxidase test	**+**ve
Arginine dihydrolysis	**+**ve
Urease hydrolysis	**-**ve
Citrate utilization	**+**ve
Indole production	**-**ve
Triple sugar agar	**+**ve for glucose fermentation
H_2_S production	**-**ve
Nitrate reduction	**-**ve
Methyl red	**+**ve
Voges–Proskauer	**-**ve
Starch hydrolysis	**+**ve
Gelatin hydrolysis	**+**ve
Cellulose hydrolysis	**+**ve
Lipase production	**-**ve
Anaerobic growth	Poor growth with no pigment production
Haemolysis test on blood agar	+ve
DNAse test	+ve
Growth at different pH & temperatures	Optimum growth: pH 6 to 9 No growth: below 5 and above 12 pH Optimum growth: 30 °C No growth: at 10 °C & 45 °C
*Acid formation:* sugar utilization	Fully utilized: fructose and mannose Poorly utilized: dextrose, inulin, cellobiose and xylose
*Amino acid utilization*	Arginine, ornathine and lysine
*Chemotaxonomy* Fatty acid profiling (Sim: 0.6)	C_16:1ω7c_ (32.7%), C_16:0_ (26.3%), C_18:1ω7c_ (16.9%), C_12:0_ (6.2%), C_10:0_ (4.8%) and C_12:0–3 OH_ (4.1%)
*Molecular analysis* 16S rRNA gene sequence analysis & identity	GenBank Accession MK 841034 with similarity to *C. violaceum* ATCC 12472

**Table 2:** Functional characteristics of *Chromobacterium vialoceum* strain TRFM-24 under *in vitro* conditions

Characteristics	Strain TRFM-24
ACC deaminase	-ve
IAA production	**+**ve
Siderophore production	-ve
Cellulase production	**+**ve
Xylanse production	**+**ve
Protease production	**+**ve
Salinity tolerance (NaCl)	Optimum growth at 1.0% in tryptone medium No growth on salt up to 2.5%
Drought tolerance	Tolerate osmotic pressure up to -1.2 MPa (9.3% PEG6000)
Temperature tolerance	Growth between 20 and 35 °C
Antagonistic to phytopathogens	No antagonism against any test phytopathogen
Ammonium production	**+**ve
Hydrogen cyanide production (HCN)	**-**ve
Solubilization of P, Zn, K	No solubilization

*MPa* : Megapascal

### Viability and preservation

**Fig. 3:**
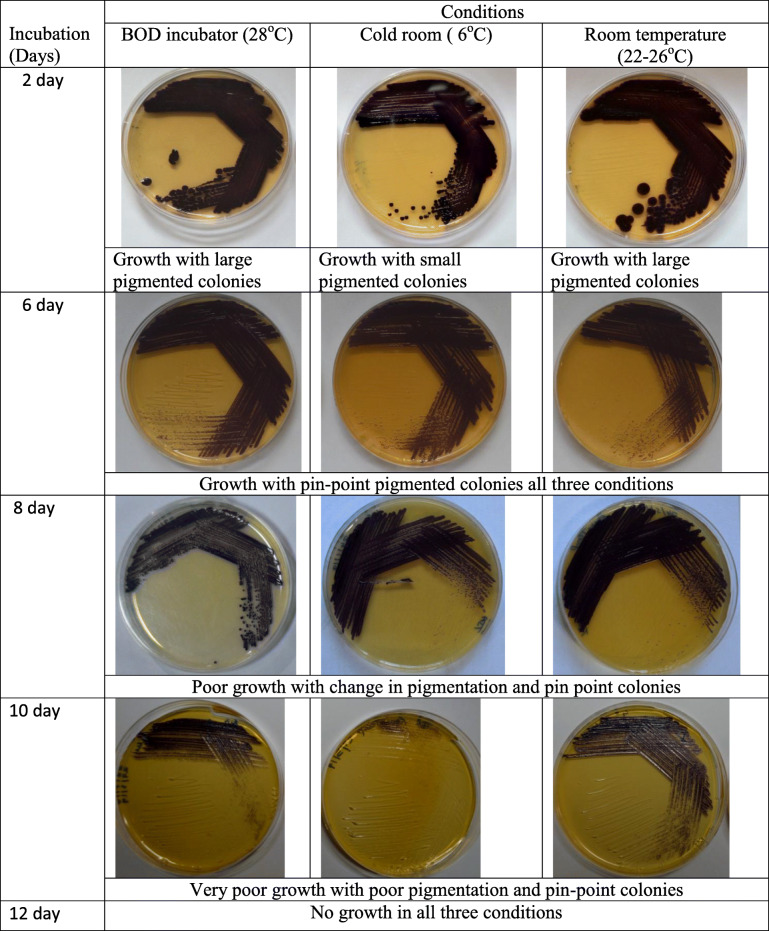
Viability status of *Chromobacterium violaceum* strain TRFM-24 in three different temperature conditions after 12 days

**Fig. 4:**
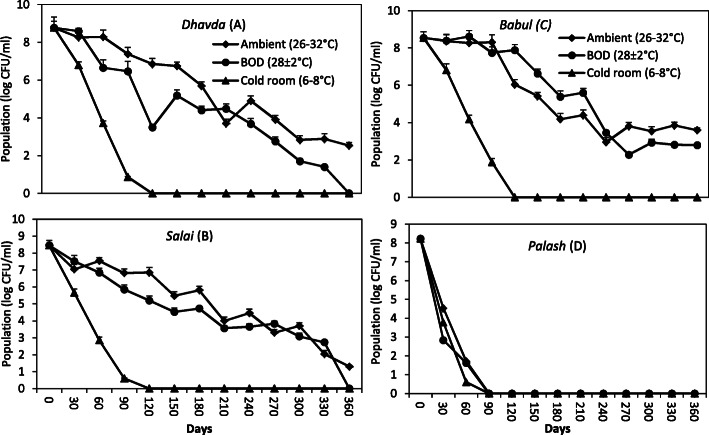
Population dynamics of *Chromobacterium violaceum* strain TRFM-24 in four different natural plant gums **a** *Dhavda*, LSD (*P = 0.05*) 0.88; **b** *Salai*, LSD (*P = 0.05*) 0.69; **c** *Babul*, LSD (*P = 0.05*) 0.83; **d** *Palash*, LSD (*P = 0.05*) 0.67 under various temperature regimes (ambient temperature (26–32 °C), BOD incubator (28 ± 2 °C) and cold room (6–8 °C) conditions; *LSD*, least significant difference (*P = 0.05*); data are mean of three replications; error bars are the standard deviation of means

**Fig. 5:**
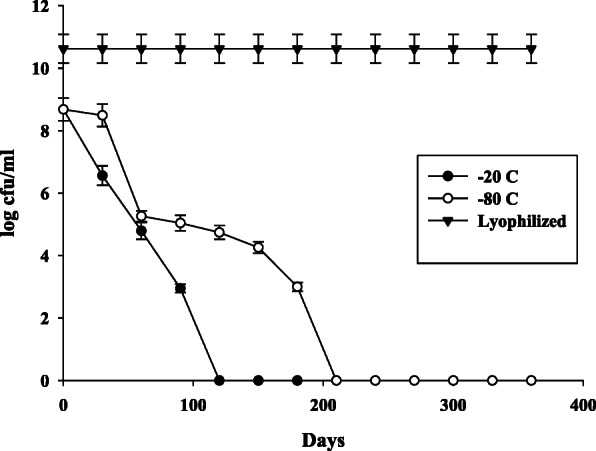
Population dynamic of *Chromobacterium violaceum* strain TRFM-24 in conventional standard preservation methods

The strain TRFM-24 was grown for 24 h followed by incubation on culture plates under three different conditions. It showed differential growth, and the culture lost its viability in all the three conditions after 12 days (Fig. 3). It was observed that the culture grown at 6–8 °C in cold room has lost viability even before 10 days possibly due to cold shock, but in the incubator and at room temperature, it survived a bit longer. However, on 12th day, the culture totally lost its viability. In order to enhance viability of the cultures, different low-cost natural gums have been used for preservation of this bacterium. Based on the three-way ANOVA results, irrespective of temperature and time, in general, out of the 4 natural gums, maximum survival of the bacterium was recorded in *babul*, *salai* and *dhavda* gums. The survival was  beyond 360 days of incubation at room temperature and to a similar extent in the BOD incubator at 28 °C temperature (Fig. 4)., In the palash gum medium, the culture did not survive after 90 days of incubation at all the three temperatures. Overall, the babul gum supported maximum survival (10^8^ CFU ml^-1^), whereas the same population was maintained from the beginning up to 90 days at room and incubator temperatures. All the gums supported survival of the bacterium up to 3 months even at 4 °C in refrigerated condition, but subsequently bacterial population declined rapidly. Besides gum-based preservation, bacterium was also stored in glycerol stock in order to analyse its survival by conventional methods. The results revealed the viability of cells up to 360 days and beyond in gums at room temperature. However, the bacterial population was drastically reduced at 4 °C. The bacterial colonies that appeared on plate after 360 days of storage in the *babul*, *dhavda* and *salai* gums were found to be violet pigmented, circular, smooth, entire and convex which is in conformity with original characteristics. However, colonies from the palash gum were viscous which is in contrast to the original characteristics. In the glycerol stock, maximum survival up to 180 days was retained at - 80 °C. However, at - 20 °C, cell viability was retained up to 120 days. Conventionally, lyophilization has been used to ensure maximum survival of the bacteria without any change in its features (Fig. 5). The above results clearly indicate that natural gums are better stabilizing agents in preserving this bacterium for 360 days and beyond at room temperature.

## Discussion

There are 25 diverse hotspots spread across the globe, out of which namely Indo-Burma and Western Ghats located in India are considered as the hottest hotspots based on endemism to plants and animals [46]. The Tripura state falls in Indo-Burma hotspot of the country. In this study, among the bacteria recovered, a violet-pigmented bacterial isolate designated as TRFM-24 was identified as *Chromobacterium violaceum* based on the polyphasic approach. In India, most of the *Chromobacterium violaceum* and *Chromobacterium* spp. have been isolated from clinical samples and a very few from the soil, water and plants [47, 48].This result supported the report that most of the *C. violaceum* of soil–plant–water origin were recovered from different regions of Amazon, Brazil. It has been observed that *C. violaceum* shows differential growth behaviour on different media which aligns with our work, wherein the strain TRFM-24 grew well on TSA, although it was isolated on Angel’s agar medium [49, 50]. The morphological, physiological and biochemical features data generated for the strain TRFM-24 in this study are matching, with some exception, with the features of *C. violaceum* strains ATCC 12472, YM1, CVAC7-1, CVRP27-1, CV5, CV 10 and CV17 isolated from different soil and water sources from various countries [4, 51, 52]. Our strain exhibited haemolysis on blood agar and was DNAase positive which is indicative of possible pathogenicity to human beings and is in line with traits present in other strains reported [53, 54]. The strain TRFM-24 was also found to be negative for indole production which supports report of Corpe [55]. Production of indole by any strain of this bacterium may open a new avenue to study tryptophan-independent pathway for IAA production.

In terms of functional traits, the strain TRFM-24 possesses only a few important plant growth-promoting traits (IAA, cellulase, xylanase, ammonium production, etc.) and did not show any antagonism towards phytopathogens. This is in contrast to a report that some of the *Chromobacterium* strains from Brazil and USA exhibited antagonism against beneficial microbes and phytopathogens by being able to produce cyanide, chitinolytic enzymes and release of volatile organic compounds (VOC) [4, 56, 57]. However, the strain TRFM-24 does not produce  cyanide which is in contrast to other strains of *C. violaceum* reported elsewhere [58]. The strain TRFM-24 has the least number of plant growth–promoting traits as compared to the most widely used plant growth–promoting rhizobacteria like *Bacillus* and *Pseudomonas* [59, 60].

The strain TRFM-24 had a short lifespan of 10 days at 4–10 °C and at room temperature and BOD incubator which supported the earlier work, wherein sensitivity of *C. violaceum* to low (1–2 days or 4 °C) temperature and also at 12 °C was reported [4, 61]. To preserve this bacterium at room temperature, we have developed a bacterial preservation process involving low-cost, water-soluble natural gums namely *babul* (acacia gum), *dhavda* and *salai* to overcome low-temperature stress to increase survivability of the strain TRFM-24. The long-term survivability due to low temperature may be governed by predominance of arabinose and other components in most of the gums (Table 3). In general, maximum survival of this bacterium was recorded in *babul*, *salai* and *dhavda* gums after 360 days of incubation at room temperature and to a similar extent in the BOD incubator at 28 ± 2 °C temperature, whereas in the *palash* gum, the culture did not survive after 90 days of incubation at all three temperatures. The potential of gum acacia in the preservation of *E. coli*, *Bacillus subtilis*, *B. anthracis*, *B. thuriengiensis*, *Lactobacillus* and *Beijerinkia* for a substantial period of time has already been documented [24, 25, 62, 63, 64]. However, there is no report of use of natural gums *dhavda* and *salai *for extending the survivability of the above said bacteria. The possible reason for the protection and preservation of this bacterium might be attributed to the conferment of structural integrity, reduction in metabolic stress and slowdown of metabolic processes by carbohydrate components of these gums. In contrast, the *palash* gum did not preserve this bacterium for a longer period, unlike other gums, possibly due to presence of tannins which might adversely affect viability of bacteria by damaging the membrane, inhibiting extracellular enzymes, deprivation of substrate required for growth and inhibition of microbial metabolisms by affecting oxidative phosphorylation [65, 66]. Such preserved *Chromobacterium* might be used as a biosensor to detect tryptophan, vitamin B12 and biochemical oxygen demand (BOD) in environmental, agricultural, medical, soil–water and fermentation samples [67, 68, 69]. In one of the previous studies, it has been noticed that formation of violacein pigment by the strain TRFM-24 is dependent on the amount of tryptophan present in the samples [22].

**Table 3:** Composition of natural gums and their solubility in water and organic solvents

Name of natural gums [moisture retention (%)]	Composition	Solubility	References
*Babul *gum (acacia gum) [*Acacia nilotica* (*7.32%*)]	Protein, rhamnose, arabinose, galactose and uronic acid	Water and other organic solvents	[70]
*Palash* or *kamarkas *gum [*Butea monosperma* (13.6%)]	Tannin with mucilaginous material and pyrocatechin	Sparingly soluble in water but highly soluble in organic solvent	[71]
*Dhavda *gum (India gum) [*Anogeissus latifolia* (9.66%)]	Arabinose, galactose, mannose, xylose, glucuronic acid, glucose and uronic acid	Water and other organic solvents	[72]
*Salai *gum (gum resin) [*Boswellia serrata* (2.36%)]	Arabinose, galactose, xylose, resin (30–60%)	Water and other organic solvent	[73]

*Chromobacterium* species occur in the natural soil–water environment of tropical and sub-tropical areas and is sensitive to low temperature. It is now assumed that with the effect of global warming, the geographic distribution of *Chromobacterium* is more at the global level as compared to  previous  concentration in the northern hemisphere only [74]. Such  increasing  trend of *Chromobacterium* spp across the globe may be more devastating. Hence, it would become a challenge and needs special attention in order to develop suitable strategies to treat-to-difficult pathogen.

## Conclusion

*Chromobacterium violaceum* TRFM-24 of the rice rhizosphere possesses typical features such as ability to grow luxuriantly on TSA at 28 °C; tolerance to salinity (2.5% NaCl) and drought (-1.2 MPa); ability to produce cellulase, xylanase and protease; inability to produce indole, hydrogen cyanide, and ACC deaminase; no antagonism towards any major phytopathogens; and inability to solubilise Zn, P, K. Since *C. violaceum* TFRM-24 survived for not more than 10 days, a simple cost-effective method was developed using 1.5% aqueous suspension of natural gums to preserve this bacterium at room temperature. Among the gums, *babul* (gum acacia), *dhavda* and *salai* preserved this bacterium for 12 months and beyond at room temperature. Perhaps, this is the first report of preservation of *C. violaceum* in natural gums at room temperature without involving any sophisticated infrastructure. Further, this preservation technique may be used by researchers for facilitating more research on this bacterium in the field of agriculture, biotechnology, industry, clinical and medical sciences.

## Supplementary Information

**Additional file 1: Supplementary Fig. 1** Soil sampling sites in Tripura state of India (Row 1: site from where strain TRFM-24 was isolated)

**Additional file 2: Supplementary Table 1** Different *Chromobacterium* species with their niches

## Data Availability

All data generated or analysed during this study are included in this published article.

## Abbreviations

NAIMCC: National agriculturally important microbial culture collection
MCC: Microbial culture collection
IAA: Indole-3-acetic acid
TSA: Tryptic soya agar
NRRL: Northern regional research laboratory
DMSO: Dimethylsulfoxide
NA: Nutrient agar
KB: Kings B
BHI: Brain heart infusion
H_2_S: Hydrogen sulphide
LBA: Luria-Bertani agar
FAME: Fatty acid methyl esters
GC: Gas chromatrography
MIDI: Sherlock microbial identification system
WDCM: World data centre for microorganisms
IDA: International depository authority
TSB: Tryptic soya broth
MH: Mueller hinton
ACC: 1-Aminocyclopropane-1-carboxylic acid
FeCl_3_: Ferric chloride
CAS: Chrome azurol S
DF: Dworkin Foster
PDA: Potato dextrose agar
PEG: Polyethylene glycol
HCN: Hydrogen cyanide
MPa: Megapascal
BOD: Biological oxygen demand
CRD: Complete randomised design
OD: Optical density
CFU: Colony-forming unit
ANOVA: Analysis of variance
LSD: Least significant difference
rRNA: Ribosomal ribonucleic acid
ATCC: American type culture collection
VOC: Volatile organic compounds
