# Supplementary figures and images for: Characterization and low-cost preservation of Chromobacterium violaceum strain TRFM-24 isolated from Tripura state, India
Source: J Genet Eng Biotechnol. 2021 Oct 1;19:146. doi: 10.1186/s43141-021-00241-z (PMC8486904; doi:10.1186/s43141-021-00241-z)

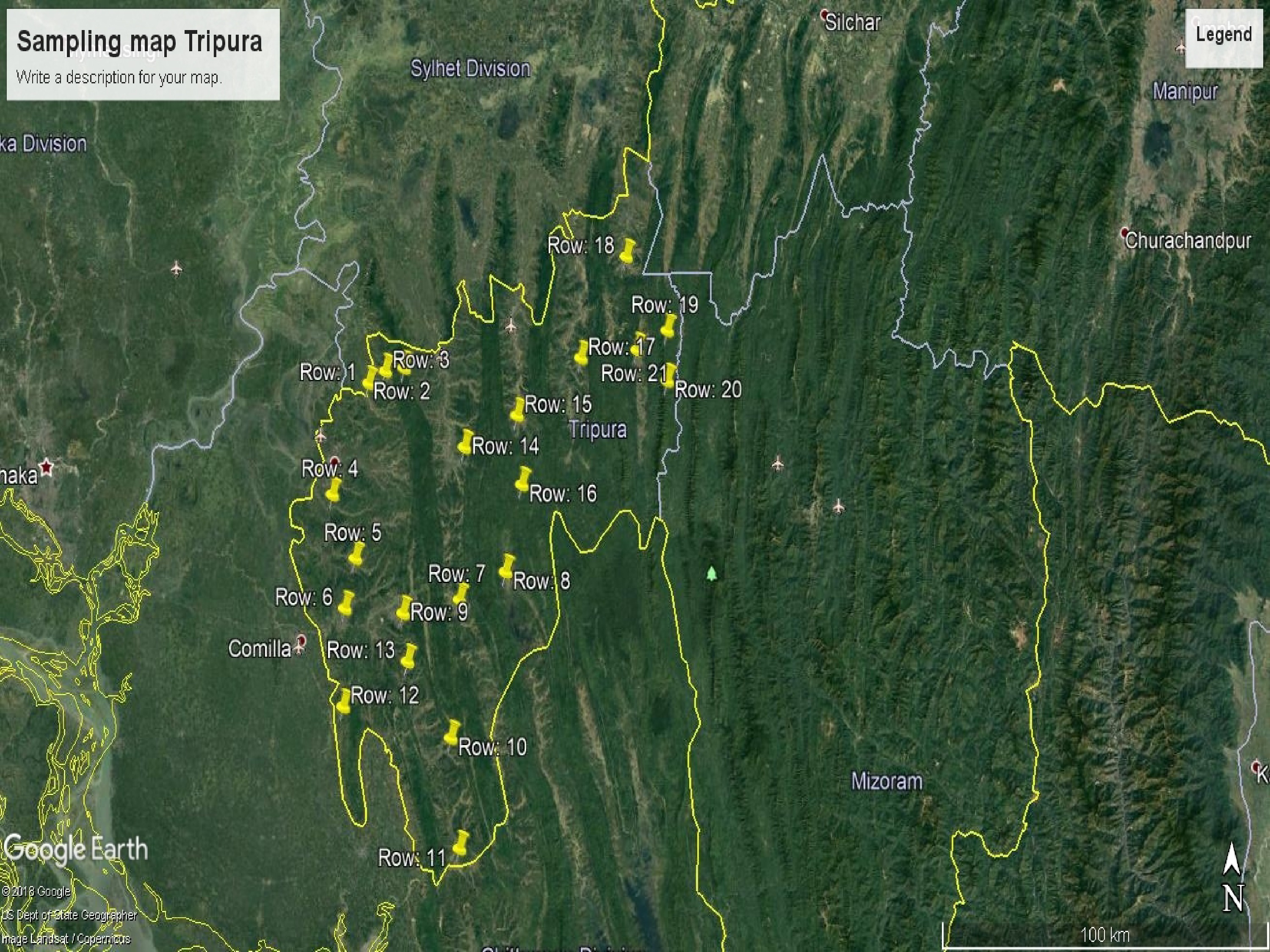

Supplement: Supplementary file 1 — Additional file 1: Supplementary Fig. 1 Soil sampling sites in Tripura state of India (Row 1: site from where strain TRFM-24 was isolated) [file 43141_2021_241_MOESM1_ESM.jpg]
