# Supplementary material for: Characterization and low-cost preservation of Chromobacterium violaceum strain TRFM-24 isolated from Tripura state, India
Source: J Genet Eng Biotechnol. 2021 Oct 1;19:146. doi: 10.1186/s43141-021-00241-z (PMC8486904; doi:10.1186/s43141-021-00241-z)
Supplement: Supplementary file 2 — Additional file 2: Supplementary Table 1 Different Chromobacterium species with their niches [file 43141_2021_241_MOESM2_ESM.docx]

**Supplementary Table 1** Different *Chromobacterium* species with their niches

| Species/strains | Source of isolation |  |
| --- | --- | --- |
| *Chromobacterium violaceum* | Wet rice paste |  |
| *C.lividum* reclassified as Janthinobacterium lividum | Not known |  |
| *C. iodinum* reclassified as *Brevibacterium iodinum* | Milk |  |
| *C. marismortui* ATCC 17056^T^reclassified as *Chromohalobacter marismortui* | Dead sea |  |
| *C. marinum* | Open ocean water |  |
| C.fluvitatile reclassified as *Idobacter* fluvitatile | Water |  |
| *C. subtsugae* PRAA4-1^T^ | Maryland forest soil |  |
| *C. aquaticum* CC-SEYA-1^T^ | Spring-water samples |  |
| *C. haemolyticum*  MDA0585^T^ | Sputum culture of patient |  |
| *C. piscinae* LMG 3947^T^ | Environmental samples |  |
| *C. pseudoviolaceum*  LMG 3953^T^ | Environmental samples |  |
| *C. vaccinii MWU205^T^* | Soil and root of cranberry |  |
| *C. amazonense* CBMAI 310^T^ | Water sample |  |
| *C. alkanivorans* ITR-71^T^ | Pesticide contaminated soil |  |
| *C. rhizoryzae* LAM1188^T^ | Rice roots |  |
| *C. sphagni* IIBBL 14B-1^T^ | *Sphagnum* bogs |  |
| *C. phragmitis* IIBBL 112-1^T^ | Estuarine marshes |  |
